# Pulmonary lymphangioleimyomatosis and systemic lupus erythematosus in a menopausal woman

**DOI:** 10.1186/s12882-018-0889-2

**Published:** 2018-04-18

**Authors:** Hong Hong, Ruiheng Yang, Xiuzhen Li, Mengjun Wang, Zhongchao Ma

**Affiliations:** Department of Nephrology, Liao Cheng People’s Hospital, No. 67 West Dongchang Road, Dongchang District, Liaocheng, Shandong Province People’s Republic of China

**Keywords:** Lymphangioleimyomatosis, Lung, Systemic lupus erythematosus, Menopausal

## Abstract

**Background:**

Pulmonary lymphangioleimyomatosis (PLAM) is a rare disease involving lung. PLAM primarily affects young women, a characteristic it shares with systemic lupus erythematosus (SLE). Estrogen has long been assumed to play an important role both in PLAM and SLE. We report a menopausal woman, who was found to have PLAM 1 year after she was diagnosed with SLE. Her chest radiograph was normal in the early phase of SLE.

**Case presentation:**

A 52-year-old Chinese woman was referred to our hospital in August 2014 because of swelling in both legs. She also reported a malar rash and intermittent generalized arthralgia. Laboratory examination showed leukopenia. Her serum albumin level was 23 g/L; 24-h urinary protein excretion was 5.3 g. She tested positive for anti-Smith (Sm) antibody and anti-SS-A antibody. Renal biopsy indicated Class V + IV(G)-A lupus nephritis (LN). The condition of SLE and LN improved on a regime of tapering prednisolone and intermittent intravenous cyclophosphamide therapy until 1 year later when she developed exertional dyspnea accompanied with frequent cough. Thoracic computed tomography revealed numerous well-defined cysts and the diagnosis of PLAM was confirmed by lung biopsy. In the follow-up period, the patient continued to be on prednisolone and mycophenolate mofetil for the treatment of SLE, but only agreed to receive symptomatic treatment for PLAM. One year after the diagnosis of PLAM, during which time the SLE was stable, she died of respiratory failure and cor pulmonale.

**Conclusion:**

We report a patient with coexisting SLE and PLAM, who was treated with immunosuppressive therapy. SLE was stable but PLAM was not improved. Although the coexistence of SLE and PLAM might be a coincidence, the occurrence of these two diseases in a menopausal woman may warrant further mechanistic exploration.

## Background

Lymphangioleiomyomatosis (LAM) is a rare condition that can affect multiple systems, but predominantly affects the lungs. The condition typically occurs in young women. Pulmonary LAM (PLAM) is characterized by diffuse proliferation and infiltration of abnormal smooth muscle cells (LAM cell) [1, 2]. The cardinal manifestations of PLAM are dyspnea on exertion and recurrent pneumothorax. Since PLAM often affects young women and typically worsens during pregnancy or following the administration of estrogens, anti-hormone therapy is assumed to be a rational approach. However, the effectiveness of this therapy is widely contested. Progressive PLAM can ultimately lead to respiratory failure. Due to the lack of effective treatment, the overall prognosis is poor in the absence of lung transplantation. However, in recent years, sirolimus has been increasingly recognized as an effective treatment for PLAM. Likewise, systemic lupus erythematosus (SLE), an inflammatory multi-system disease of unknown etiology, also predominantly affects female population. In this case report, we describe a menopausal woman with coexisting SLE and LAM. Before the diagnosis of SLE, she was affected by thyroid papillary carcinoma.

## Case presentation

A 52-year-old Chinese female farmer was referred to our hospital in August 2014 because of bilateral leg swelling since approximately 2 weeks. She had a history of malar rash and intermittent generalized arthralgia since 3 months. She also had a history of right thyroid papillary carcinoma, for which subtotal thyroidectomy was performed in 2009. Postoperatively, she was administered L-Thyroxine therapy and was followed up annually. The dosage of L-Thyroxine at admission was 50 μg daily. She was a non-smoker and had developed menopause at the age of 50 years. The family history was unremarkable. Physical examination showed a blood pressure of 130/80 mmHg, malar rash, clear breath sounds, normal heart sounds, and grade 3 lower-extremity pitting edema. There was no redness or swelling over joints. Her serum creatinine level was 1.37 mg/dL (121 μmol/L); serum albumin level was 2.3 g/dL (23 g/L); white blood cell (WBC) count was 2700/μL; lymphocyte count was 600/μL. Urinary protein excretion was 5.3 g/d. Serological tests for antinuclear antibody (ANA; 1:320, speckled pattern), anti-Smith (Sm) antibody, and anti-SS-A antibody were positive. Antineutrophil cytoplasmic antibody (ANCA), hepatitis B surface antigen, and hepatitis C antibody were negative. She had low serum complement levels (C3, C4). Anti-streptolysin O titer, and rheumatoid factor were within normal range. Urine microscopy showed 20–30 red blood cells and 3–8 WBCs per high-power field. Electrocardiogram, chest radiograph and kidney ultrasound were unremarkable.

**Fig. 1:**
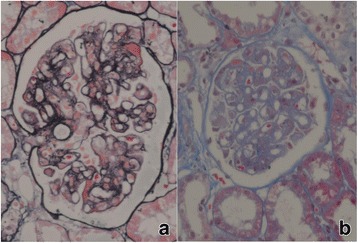
Histopathological examination of renal biopsy specimen. **a** Light micrograph of a glomerulus showing diffuse thickening of the glomerular basement membranes; with the silver stain, diffuse subepithelial silver positive projections (spikes) from the capillary basement membranes are seen. (Methenamine silver and Masson trichrome stain, × 400). **b** Light micrograph showing mesangial hypercellularity and mesangial matrix expansion. (Periodic acid Schiff stain, × 400)

Based on clinical and laboratory findings, a diagnosis of SLE, lupus nephritis (LN) and nephritic syndrome was established. One week later, a percutaneous renal biopsy was performed. Light microscopy showed that there were 38 glomeruli, with a mild increase in mesangial cellularity and mesangial matrix. There were no signs of endocapillary proliferation. Glomerular basement membranes were thickened and spikes were noticed on Jones silver staining (Fig. 1 a, b). On immunoflurescence examination, the mesangium and glomerular capillary wall were found positive for IgG (3+), IgA (1+), IgM (2+), C3 (2+), C1q (2+), and FRA (1+). Electron microscopy showed irregular thickening of the glomerular basement membranes, mesangial hypercellularity, proliferation of the mesangial matrix, and electron-dense subepithelial, intramembranous, subendothelial and mesangial deposits. These findings were consistent with Class V + IV(G)-A lupus nephritis.

**Fig. 2:**
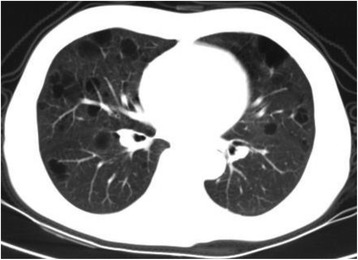
Thoracic high resolution computed tomography showing numerous well-defined thin-walled cysts

**Fig. 3:**
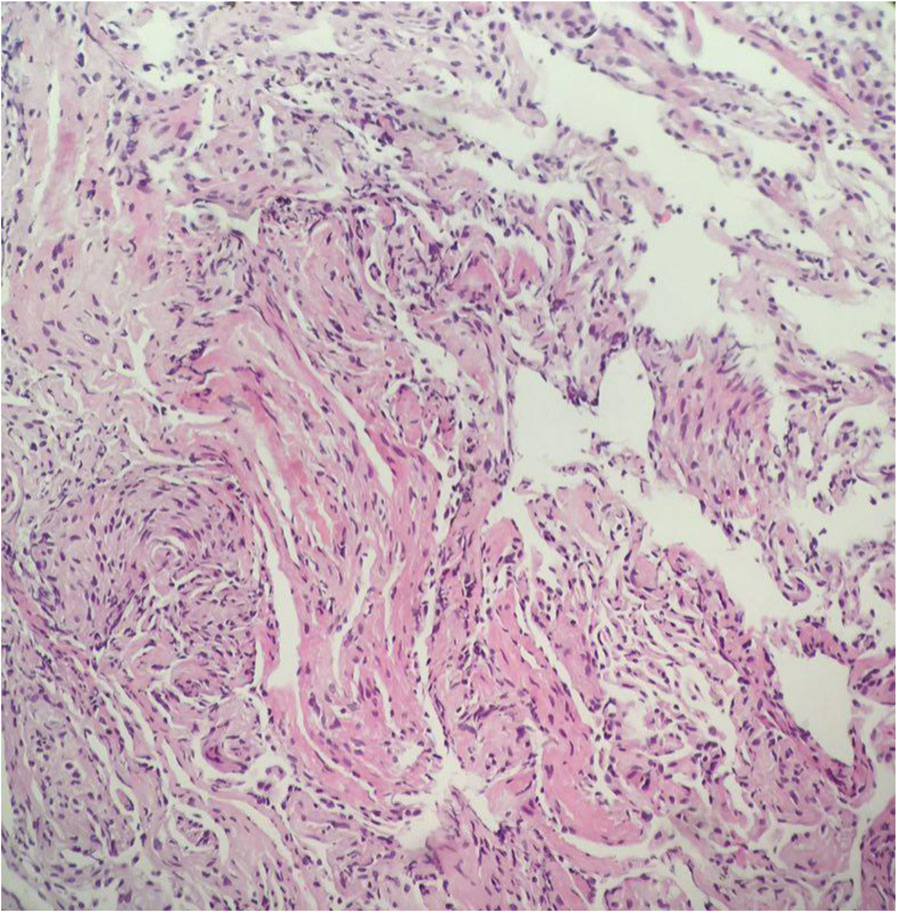
Pathological examination of Lung biopsy. The lung biopsy showed dilated cystic cavity, spindle cells were seen in the cavity wall, which appeared like smooth muscle cells (LAM cells). (HE stain, × 400)

**Fig. 4:**
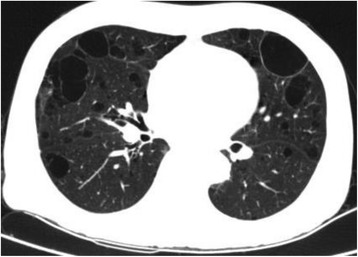
Repeat computed tomography showing multiple pulmonary cysts and bullae in both lungs, and interstitial pulmonary fibrosis

After the diagnosis, we instituted treatment with intravenous methylprednisolone 500 mg/d for 3 days, followed by 1 mg/kg oral prednisolone daily. In addition, she received omeprazole (20 mg, twice a day), rocaltrol (0.25 μg, once a day), and caltrate D (300 mg, twice a day) for symptomatic treatment. Ten days after treatment, the WBC counts were restored to normal levels. Subsequently, intermittent intravenous cyclophosphamide treatment was added. After four-week treatment, the edema was largely resolved; her serum albumin level was 2.4 g/dL (24 g/L) and her urinary protein excretion was 4.0 g/d. She was discharged from the hospital and followed up monthly. Eight months later, her urinary protein excretion was 1.2 g/d, serum creatinine level was 1.1 mg/dL (97 μmol/L), and serum albumin level was 3.0 g/dL (30 g/L). There was no recurrence of rashes or arthralgia. Her condition continued to improve for about 15 months (until November 2015), at which time, the dosage of prednisolone was 12.5 mg daily and that of total cyclophosphamide was 8 g. Then she experienced exacerbation of symptoms with exertional dyspnea and frequent cough and sputum. She was hospitalized again. Sputum culture showed streptococcus pneumoniae and thoracic computed tomography revealed numerous well-defined cysts (Fig. 2). Meanwhile, she developed recurrence of lupus nephritis. We instituted prednisolone 1 mg/kg daily combined with mycophenolate mofetil 0.75 g twice a day after the control of infection. A diagnosis of pulmonary LAM was made based on lung biopsy performed at another hospital (Fig. 3). Patient only agreed to receive symptomatic treatment, i.e., oxygen supplementation, ipratropium and acetylcysteine. The patient refused to receive sirolimus and estrogen. In the following years, lupus nephritis was stable (partial remission); her serum creatinine level was 1.45 mg/dL (128 μmol/L), serum albumin concentration was 2.9 g/dL (29 g/L), and urine protein to creatine ratio was 2.3. The dosage of oral prednisone was 20 mg daily and mycophenolate mofetil was 0.5 g twice a day. However, she experienced progressive dyspnea. CT re-examination confirmed progressive multiple thin-walled pulmonary cysts and bullae in both lungs, along with interstitial pulmonary fibrosis (Fig. 4). She died in December 2016 due to severe respiratory failure and cor pulmonale.

## Discussion

Lymphangioleiomyomatosis predominantly affects young women, and is rarely reported in menopausal women or in men. It primarily affects the lungs; involvement of other organs such as lymphatic system, mediastinum, liver, uterus, pancreas, and kidneys has also been reported. Owing to its rarity, LAM is liable to misdiagnos is; a delay in diagnosis of 28–44 months has been reported [3]. Nearly all patients have dyspnoea at the time of diagnosis [4, 5]. In the early stage, chest radiographic findings may be normal. However, high-resolution computed tomography findings are almost always abnormal at the time of diagnosis and can be a more sensitive indicator of early disease in patients who have normal chest radiographs [6]. Standard chest radiographs are of limited diagnostic utility owing to their poor sensitivity. The natural history of PLAM is one of progressive airflow obstruction. Available treatment options include progesterone, sirolimus, tamoxifen and reduction of oestrogen exposure (e.g., by bilateral oophorectomy). Of note, sirolimus has been shown to stabilize lung function, reduce serum VEGF-D levels, and is associated with a reduction in symptoms and improvement in quality of life [7]. The reported 5-year survival rates range between 50% and 97% [8, 9].

SLE, an autoimmune disorder, predominantly affects female population and can affect nearly all organs. The most common respiratory manifestation of SLE is pleural disease (occurs in approximately 50% of all patients), which may present as pleural effusion [10, 11]. Interstitial lung disease is a less commonly reported finding in SLE as compared to that in other connective tissue disorders [12]. There is a higher incidence of pulmonary embolism in those with antiphospholipid syndrome. Immunosuppressive medications may predispose patients to opportunistic pathogens as well as to community-acquired pneumonia. PLAM, as a respiratory manifestation of SLE, has never been reported in the literature. To the best of our knowledge, L-Thyroxine has not been reported to worsen the disease course of SLE and LAM.

In the present case, the patient had no subjective respiratory symptoms at the time of initial diagnosis of SLE, and the chest radiograph was normal. Though these manifestations could not exclude PLAM at that time, it was not indicative of a progressive disease. In the following 2 years, the patient was treated with continuous immunosuppressive therapy, during which time the SLE was stable and LN was partly resolved. Irrespective of the time of development of PLAM during the stable stage of SLE, this disease showed a rapidly progressive course in the last 2 years and did not respond to immunosuppressive therapy. Although estrogen had long been assumed to play an important role in both LAM and SLE, this patient was a menopausal woman, and the development of these two diseases did not occur concomitantly, which indicates other potential etiologies of LAM. In the last few years, mammalian target of rapamycin (mTOR) pathway has been shown to play a key role in the pathogenesis of SLE. There is also emerging evidence of a strong association between mutations of tuberous sclerosis complex (TSC) genes (TSC1 and TSC2), activation of the mTOR pathway and development of lymphangiomatosis (LAM). Hence activation of mTOR pathway may be a converging pathway in the pathogenesis of both SLE and LAM, which suggests that patients with SLE may be at risk of or susceptible to the development of LAM. Thus, the association of SLE and LAM may not just be a coincidence but may be linked by a similar pathomechanism with potential therapeutic implications for management of patients with both SLE and LAM using mTOR pathway inhibitors. At the advanced stage, patients may develop pneumonia and related acute respiratory distress syndrome, which is reported to have a high mortality rate [13, 14].

## Conclusion

In conclusion, LAM is rare and sporadic disease. It can also affect menopausal woman and immunosuppressive therapy may not prevent the development of this disease. There is no strong evidence that SLE could exacerbate the disease. LAM is a treatable disease, and appropriate diagnosis and treatment can provide notable benefit to patients. However, development of progressive exertional dyspnea marks the onset of advanced disease which is associated with a poor prognosis. Although the coexistence of SLE and LAM may be a coincidence, the occurrence of these two diseases in one patient may warrant further mechanistic exploration.

## Abbreviations

ANA: Antinuclear antbody
ANCA: Antineutrophil cytoplasmic antibody
CT: Computed tomography
FRA: Fibrin-related antigen
Ig: Immunoglobulin
LAM: Lymphangioleimyomatosis
LN: Lupus nephritis
PLAM: Pulmonary lymphangioleimyomatosis
SLE: Systemic lupus erythematosus
